# Structural and mechanistic divergence of the small (p)ppGpp synthetases RelP and RelQ

**DOI:** 10.1038/s41598-018-20634-4

**Published:** 2018-02-01

**Authors:** Wieland Steinchen, Marian S. Vogt, Florian Altegoer, Pietro I. Giammarinaro, Petra Horvatek, Christiane Wolz, Gert Bange

**Affiliations:** 1 Philipps-University Marburg, LOEWE Center for Synthetic Microbiology & Department of Chemistry, Hans-Meerwein-Straße, 35043 Marburg, Germany; 2 University of Tübingen, Interfaculty Institute of Microbiology and Infection Medicine, Elfriede-Aulhorn-Straße 6, 72076 Tübingen, Germany

## Abstract

The nutritional alarmones ppGpp and pppGpp (collectively: (p)ppGpp) are nucleotide-based second messengers enabling bacteria to respond to environmental and stress conditions. Several bacterial species contain two highly homologous (p)ppGpp synthetases named RelP (SAS2, YwaC) and RelQ (SAS1, YjbM). It is established that RelQ forms homotetramers that are subject to positive allosteric regulation by pppGpp, but structural and mechanistic insights into RelP lack behind. Here we present a structural and mechanistic characterization of RelP. In stark contrast to RelQ, RelP is not allosterically regulated by pppGpp and displays a different enzyme kinetic behavior. This discrepancy is evoked by different conformational properties of the guanosine-substrate binding site (G-Loop) of both proteins. Our study shows how minor structural divergences between close homologues result in new functional features during the course of molecular evolution.

## Introduction

Microorganisms are able to cope with a broad variety of environmental challenges such as nutrient limitation, antibiotics or changes in abiotic factors like varying pH values or temperatures. To do so, they adapt their metabolism at many different dogmatic processes, e.g. replication, transcription, translation and ribosomal biogenesis^1–3^. The ‘stringent response’ (SR) is highly conserved among bacteria^4–6^ and plant chloroplasts^7–9^ and although historically only referring to the adaptation to nutrient depletion^10,11^ it has since also been demonstrated to affect virulence^2,12,13^, biofilm formation^14^, development of cellular heterogeneity^15,16^. Moreover, in some microorganisms the SR has been suggested to affect persister cell formation^17–19^. Central to the stringent response are the two unusual nucleotides ppGpp and pppGpp (collectively (p)ppGpp or alarmones). Proteins of the RelA/SpoT homology (RSH) superfamily^20^ catalyze the pyrophosphate transfer from ATP onto the 3′-OH group of GDP or GTP, yielding ppGpp or pppGpp, respectively.

**Figure 1:**
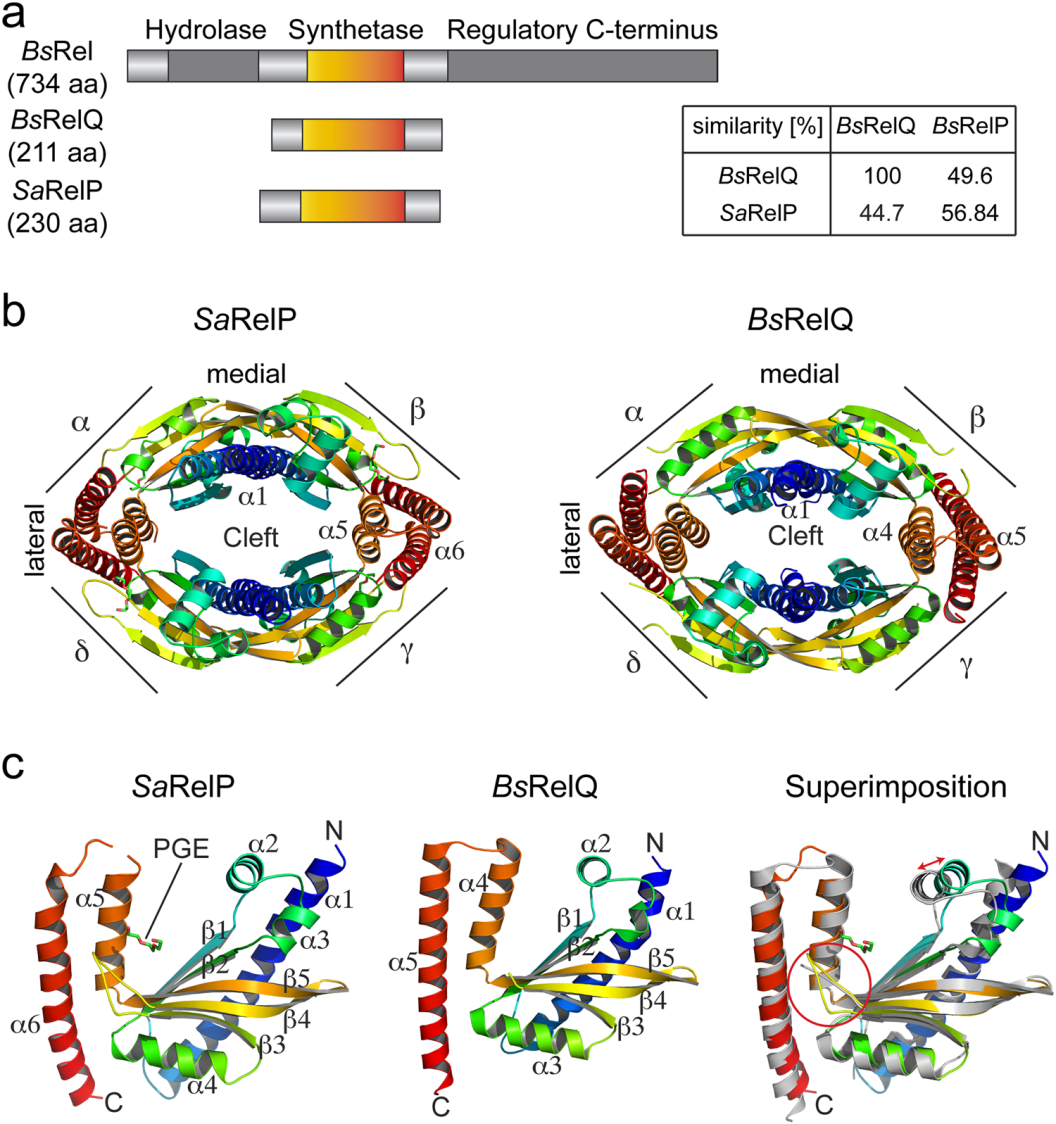
Structural analysis of RelP. (**a**) Domain architecture of the (p)ppGpp synthetases *Bs*Rel, *Bs*RelQ and *Sa*RelP drawn to scale. The inset depicts amino acid similarities between RelP and RelQ proteins from *Bacillus subtilis* (*Bs*) and *Staphylococcus aureus* (*Sa*). (**b**) Cartoon representation of the crystal structures of the *Sa*RelP (*this study*) and *Bs*RelQ (PDB: 5DEC^33^) homotetramers. Each monomer (α-δ) is rainbow-colored from its N- to its C-terminus. (**c**) The (p)ppGpp synthetase monomers of *Sa*RelP (*left; this study*), *Bs*RelQ (*middle*; PDB: 5DEC^33^) and their superimposition (*right*) coloured in rainbow from N- to C-terminus.

RSH-type synthetases fall into the two classes of ‘long’ and ‘short’ RSH (Fig. 1a^1,20^). Long RSH-type synthetases are typically composed of multiple domains and harbor a (p)ppGpp hydrolase followed by a (p)ppGpp synthetase domain in their N-terminal part (NTD). Their C-terminal portion (CTD) is highly variable and comprises domains involved in the binding of ribosomes and regulation of the opposing activities found within the NTD^21–24^. In contrast, short RSH-type alarmone synthetases only contain a synthetase domain and lack the hydrolase domain as well as regulatory domains found within the CTD of long RSH proteins (Fig. 1a). Members of this ‘small alarmone synthetase’ (SAS) family fall into the RelQ (also: SAS1) and RelP (also: SAS2) subclasses and are found in a wide range of bacteria including *Bacillus subtilis*, *Staphylococcus aureus*, *Enterococcus faecalis* and *Listeria monocytogenes*^20,25–30^. Furthermore, there is evidence for a third class of SAS proteins named RelV in *Vibrio cholerae*^31^. Noteworthy, SAS proteins typically occur in pairs (RelP and RelQ) in the same organism. Nevertheless, despite being highly similar on the amino acid sequence level (Fig. 1a), RelP/RelQ proteins seem to exhibit different functional roles as evidenced from disparate transcriptional profiles and their dependence on different stress signals^25,27,32^.

So far, only RelQ from *B. subtilis* and *Enterococcus faecalis* have been functionally characterized^29,30,33^. *Bs*RelQ shares the conserved synthetase fold with the long RSH Rel, but in contrast to the monomeric Rel, *Bs*RelQ forms highly symmetric homotetramers. Clarification of the catalytic mechanism of *Bs*RelQ showed that the enzyme binds ATP and GDP/GTP in a sequential order with ATP being the first substrate and arranges them in a near-attack conformation within the active site to catalyze immediate pyrophosphate transfer. A remarkable feature of the *Bs*RelQ homotetramer is the presence of a pronounced cleft in its center providing the binding site for two allosteric pppGpp molecules that, when present, elevate the (p)ppGpp synthetase activity of *Bs*RelQ^33^. Up to date, no structural characterization of RelP proteins is available. Also, it is unknown whether the (p)ppGpp synthesizing activity of RelP is subject to regulation. Therefore, we set out to provide a structural and biochemical comparison of RelP/RelQ proteins that might explain their divergent functional roles in bacteria.

## Results

### RelP and RelQ share an equal architecture

To better understand RelP at the molecular level, we determined the crystal structures of RelP homologues from *S. aureus* (*Sa*) and *B. subtilis* (*Bs*) at 2.25 and 3.3 Å resolution, respectively (Table S1). Both, *Sa*RelP and *Bs*RelP form highly symmetrical and oval-shaped homotetramers with a prominent cleft in their centers highly reminiscent of *Bs*RelQ (Figs 1b and S1a). Helix α1 at the N-terminus of each monomer stabilizes the medial sides of the homotetramer interface via hydrogen bonds and salt bridges (buried surface area of ~1200 Å^2^). Helices α5 and α6 at the C-terminus of each monomer establish the lateral sides of the homotetramer interface mainly due to polar contacts (buried surface area of ~1200 Å^2^). The (p)ppGpp synthetase monomers of *Sa*RelP and *Bs*RelP are highly identical and consist of a mixed β-sheet build by five β-strands (β1–β5) that is surrounded by alpha helices (α1–α6, Figs 1c and S1b).

Structural comparison of RelP and RelQ reveals the architecture of the homotetramer as well as each of the monomers is highly similar (r.m.s.d. of 1.292 over 138 Cα atoms for the RelP and RelQ monomers). However, RelP and RelQ differ in the orientation of helix α2, which appears to be shifted approximately 3 Å towards the active site center in RelQ when compared to RelP (Fig. 1c; *right panel*). Another interesting observation is that the loop connecting β3 and β4, which is disordered in the structure of *Bs*RelQ, could be resolved in both structures of RelP (Fig. 1c). Taken together, RelP and RelQ share highly conserved ternary and quaternary structures, but also reveal subtle differences that might be of functional relevance (see below).

### RelP and RelQ differ in their (p)ppGpp synthetase activity

**Figure 2:**
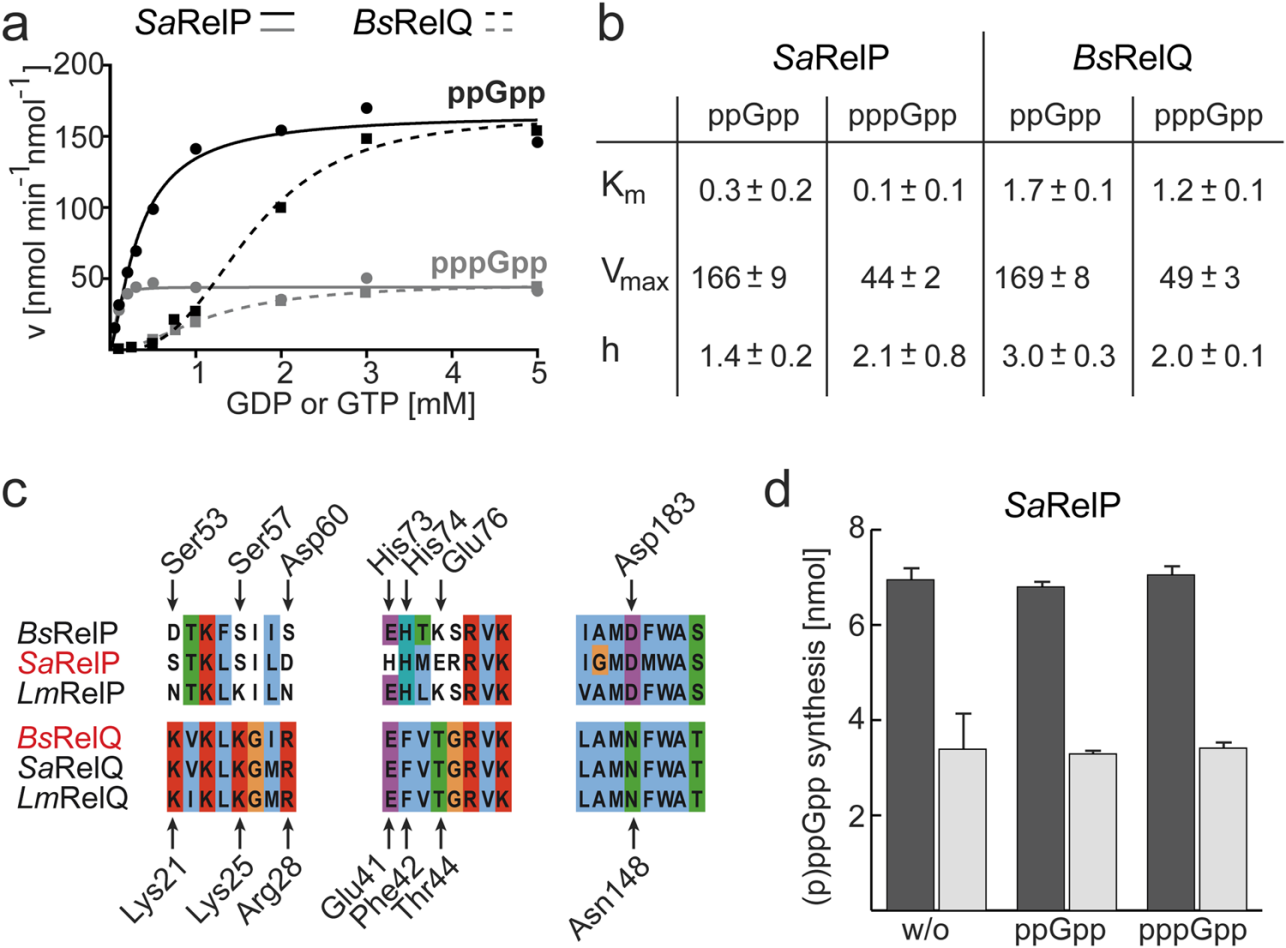
Enzymatic properties of RelP. (**a**) Velocity/substrate (v/S) characteristic of *Sa*RelP (solid lines) and *Bs*RelQ (dashed lines) for ppGpp (black) and pppGpp (grey). Velocity is given in nmol per minute per nmol *Sa*RelP/*Bs*RelQ. The data for *Bs*RelQ have been re-plotted from^33^ to enable direct comparison of both enzymatic activities. Data of one representative experiment are shown. (**b**) Kinetic parameters of (p)ppGpp synthesis by *Sa*RelP and *Bs*RelQ. (**c**) Amino acid sequence alignment of residues conferring pppGpp binding to the allosteric cleft of RelQ and their equivalent positions in RelP proteins from *Bacillus subtilis* (*Bs*), *Staphylococcus aureus* (*Sa*) and *Listeria monocytogenes* (*Lm*). Amino acid numberings relate to *Sa*RelP (above) and *Bs*RelQ (below). (**d**) Synthesis of ppGpp (black) and pppGpp (grey) by *Sa*RelP is unaffected by the presence of ppGpp or pppGpp. Error bars indicate the SD of three independent replicates.

The most distinguished features of *Bs*RelQ lie in the apparent positive cooperativity of (p)ppGpp synthesis and its susceptibility to allosteric stimulation of by pppGpp but not ppGpp^33^. To test whether both features would also be present in RelP, we performed an *in-depth* kinetic analysis. We used the same buffer composition for characterization of *Sa*RelP as previously for *Bs*RelQ to ensure maximal comparability. *Sa*RelP was incubated together with 5 mM ATP and varying concentrations of GDP or GTP (Of note: *Bs*RelP exhibited no (p)ppGpp synthetase activity under our assay conditions for unclear reasons). *Sa*RelP synthesized ppGpp more efficiently than pppGpp as evidenced from an approximately 4-fold higher V_max_ value (Fig. 2a and b). A similar preference for the product ppGpp was previously observed for *Bs*RelQ^33^ and RelQ from other organisms^25,30^. However, the K_m_ values for (p)ppGpp synthesis drastically differ between both enzymes in that they are significantly lower for *Sa*RelP (i.e. 0.3 ± 0.2 for GDP and 0.1 ± 0.1 for GTP) than for *Bs*RelQ (i.e. 1.7 ± 0.1 for GDP and 1.2 ± 0.1 for GTP; Fig. 2b). It also seemed to us that *Sa*RelP monomers displays less cooperativity within the tetramer than *Bs*RelQ indicated by Hill coefficients closer to 1 (Fig. 2b).

Amino acid sequence analysis of RelP shows that the amino acid residues required for allosteric binding of pppGpp to RelQ are replaced in RelP proteins (Fig. 2c). Indeed, this different set of amino acids found in *Sa*RelP seems incapable to coordinate pppGpp in similar fashion as *Bs*RelQ (Fig. S2) strongly suggesting to us that *Sa*RelP cannot be allosterically stimulated by the alarmone. In agreement with our structural analysis, no change in the enzymatic activity of *Sa*RelP was observed in the absence and the presence of ppGpp or pppGpp (Fig. 2d). Taken together, RelQ and RelP do not differ much in their V_max_ values of (p)ppGpp synthesis, while significantly differing in the in K_m_ values. Moreover, RelP is not subject to allosteric stimulation by pppGpp.

### ATP-binding to RelP and RelQ is identical

To gain further insights into the disparate enzymatic activities of RelQ and RelP, we attempted to solve the structure of *Sa*RelP in presence of the non-hydrolysable ATP analogue AMPCPP (α,β-methyleneadenosine 5′-triphosphate) and GDP or GTP. However, we could only obtain crystals and solve the structure of *Sa*RelP in presence of AMPCPP (Fig. S3a and Table S1). Coordination of AMPCPP within all the four active sites of *Sa*RelP is guided by π-stacking interactions of the adenine base with the arginine residues 78 and 112 of *Sa*RelP (Fig. S3b). The ribose moiety of the adenosine is coordinated by hydrogen bonding via His190. Interactions with the phosphate moieties of AMPCPP are mainly established by lysine and arginine residues residing in β1 and α2 (i.e. Lys80, Lys88 and Arg91) and Ser84 contacting the 5′ α-phosphate. AMPCPP adopts a kinked conformation that is enforced by a magnesium ion coordinated by Asp107 and Glu174 (Fig. S3b). An identical conformation of AMPCPP is observed in the active site of *Bs*RelP (Fig. S3c). As all ATP-coordinating and catalytic amino acid residues are strictly conserved among RelP/RelQ proteins (Fig. S3d), we suspect a common ATP-binding mode and mode of catalysis.

### G-Loop rigidity governs the activity of RelP and RelQ

**Figure 3:**
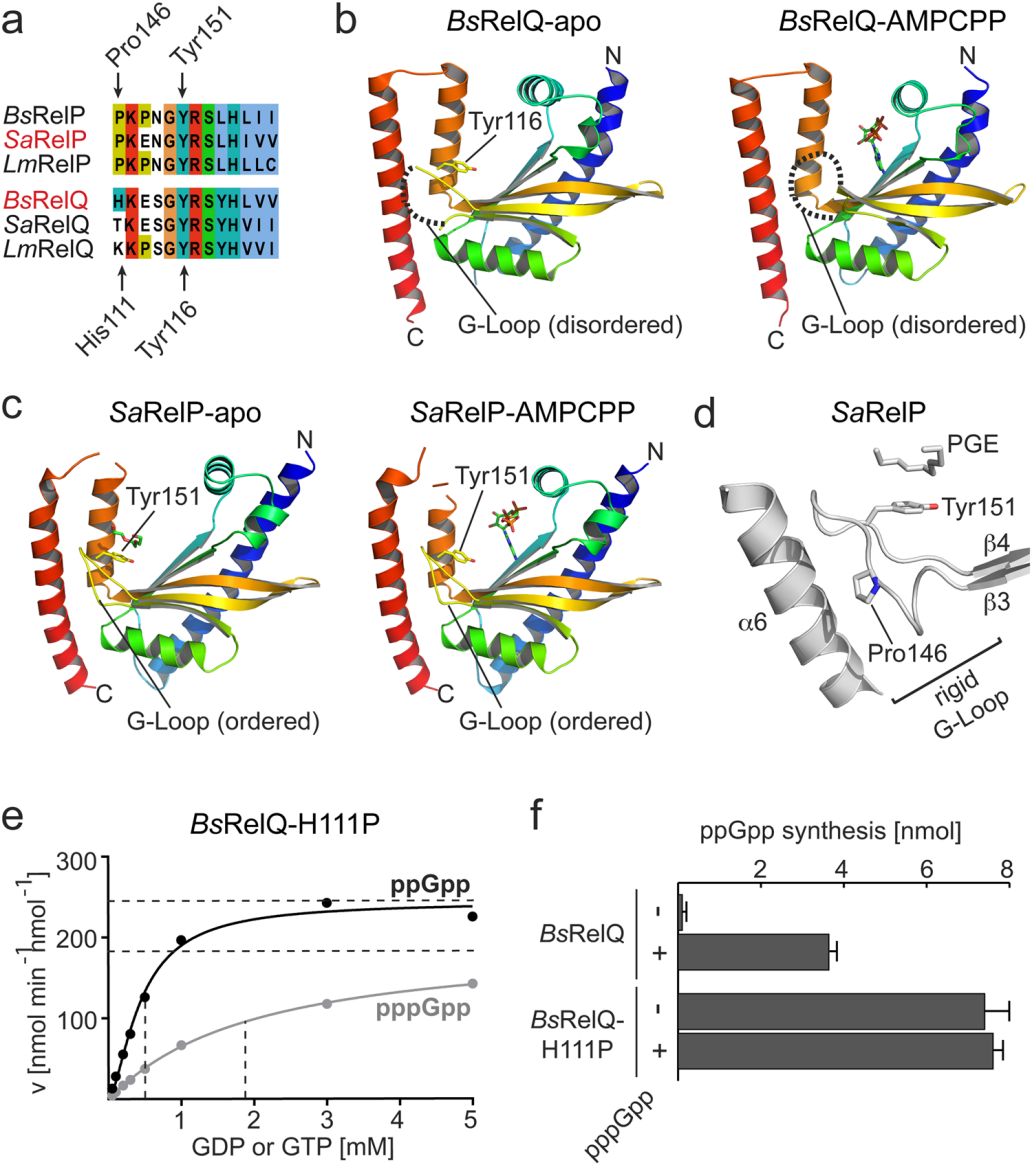
G-loop rigidity dictates the activity of RelP and RelQ. (**a**) Amino acid sequence alignment of the G-Loops found in RelP and RelQ proteins from *Bacillus subtilis* (*Bs*), *Staphylococcus aureus* (*Sa*) and *Listeria monocytogenes* (*Lm*). Amino acid numberings relate to *Sa*RelP (above) and *Bs*RelQ (below). (**b**) Crystal structures of the apo- and AMPCPP-bound state of *Bs*RelQ (PDB: 5DEC and 5F2V^33^, respectively) show a disordered G-Loop (dashed line). (**c**) Crystal structures of the apo- and AMPCPP-bound state of *Sa*RelP (this study) show a clearly ordered G-Loop. (**d**) The presence of Pro146 in *Sa*RelP confers a high rigidity of the G-Loop. (**e**) The v/S characteristic of ppGpp (black) and pppGpp (grey) synthesis of the *Bs*RelQ-H111P variant. Velocity is given in nmol per minute per nmol *Bs*RelQ-H111P. Dashed lines indicate the K_m_ and V_max_ values. Data of one representative experiment are shown. (**f**) ppGpp synthesis of *Bs*RelQ and its variants in absence (−) and presence (+) of pppGpp. Error bars indicate the SD of three independent replicates.

If binding of ATP to RelP and RelQ is identical (see above), then the different enzymatic properties of both enzymes should originate from differences in binding of GDP/GTP and/or a different susceptibility to allosteric stimulation by pppGpp. As mentioned above, our structural analysis of RelP and RelQ indicated a different conformational flexibility of the loop connecting strands β3 and β4 (Fig. 1c). This loop contains a conserved tyrosine residue (i.e. Tyr151 in *Sa*RelP and Tyr116 in *Bs*RelQ, Fig. 3a) critical to guanosine nucleotide binding in all (p)ppGpp synthetases. Therefore, we decided to term the loop connecting β3 and β4 ‘G-Loop’. To our surprise, the different configurations of the G-Loop seem to be a common theme among RelP/RelQ proteins. In the apo- and ATP-bound states of *Bs*RelQ, the G-Loop is disordered, and could therefore not be modeled in these structures (Fig. 3b). In stark contrast, the G-Loop of *Sa*RelP was well-ordered and could be unambiguously modeled in its apo- and ATP-bound structures (Fig. 3c). We speculated that the difference in enzymatic activity between RelQ and RelP is founded in the different conformational properties of the G-Loop.

Inspection of the amino acids of the G-Loop reveals the presence of proline in RelP proteins with no correspondent in RelQ (Fig. 3a). We hypothesized that the absence of this proline in RelQ renders the G-Loop less rigid, while its presence in RelP results in a well-ordered G-Loop that might easily facilitate GDP/GTP coordination (Fig. 3d). We challenged this notion by introducing proline into the disordered G-Loop of RelQ (i.e. *Bs*RelQ-H111P). *Bs*RelQ-H111P produces (p)ppGpp as efficient as *Sa*RelP and the V_max_ (i.e. 243 ± 9 and 194 ± 8 nmol min^−1^ nmol^−1^ for ppGpp and pppGpp, respectively), K_m_ (i.e. 0.4 ± 0.2 for GDP and 1.9 ± 0.2 for GTP) and Hill-coefficient (i.e. 1.6 ± 0.2 for GDP and 1.0 ± 0.1 for GTP) of *Bs*RelQ-H111P more resemble *Sa*RelP than *Bs*RelQ (Fig. 3e and compare to Fig. 2a and b). Moreover and unlike *Bs*RelQ*, Bs*RelQ-H111P is not amenable to allosteric stimulation by pppGpp (Fig. 3f). These results demonstrate a strong dependence of RelP/RelQ activity on the rigidity of the G-Loop.

### Allosteric stimulation of RelQ by pppGpp acts via the G-Loop

**Figure 4:**
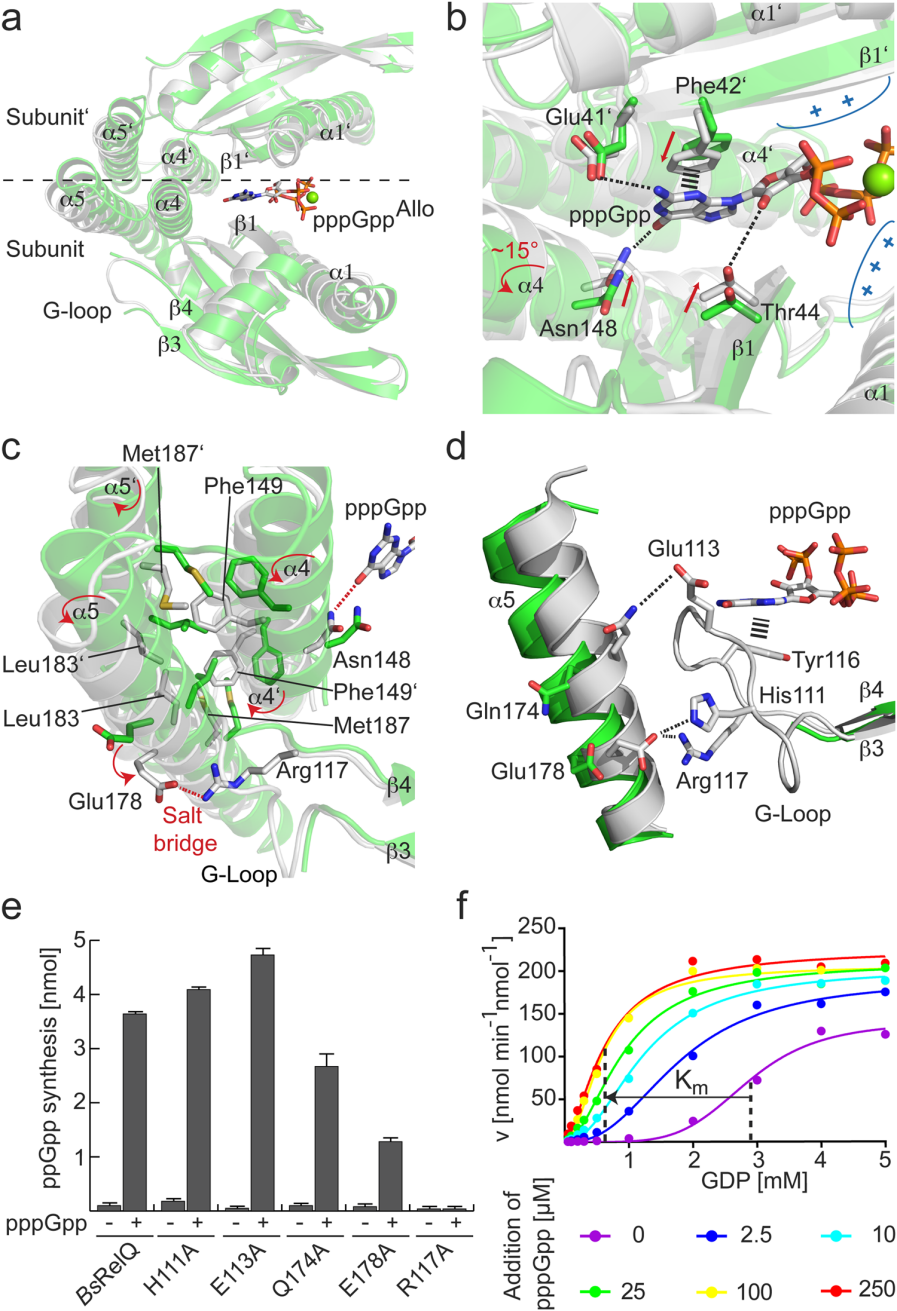
Allosteric binding of pppGpp to RelQ stabilizes the G-Loop. (**a**) Superimposition of one half of the tetramers of *Bs*RelQ (white, PDB: 5DEC^33^) and *Bs*RelQ-pppGpp (green, PDB: 5DED^33^). (**b**) Coordination of pppGpp in the central cleft of *Bs*RelQ by amino acids residing in α1, β1 and α4 results in conformational changes (indicated by red arrows). (**c**) Interaction of Asn148 with pppGpp causes a rotation of α4 that is transmitted onto α5 through the hydrophobic core established by Phe149, Leu183 and Met187 from two subunits of *Bs*RelQ. Concerted rotation of helices α4 and α5 enables formation of a salt bridge between Glu178 and Arg117. (**d**) Interactions between amino acid side chains from α5 and the G-Loop of *Bs*RelQ are only established in presence of pppGpp and result in ordering of the G-Loop. (**e**) ppGpp synthesis by *Bs*RelQ and *Bs*RelQ variants in absence (−) and presence (+) of pppGpp. Error bars indicate the SD of three independent replicates. (**f**) The v/S characteristic of ppGpp synthesis by *Bs*RelQ in presence of different amounts of pppGpp. The velocity is given in nmol per minute per nmol *Bs*RelQ. The K_m_ values of *Bs*RelQ in absence and presence of 250 µM pppGpp are indicated by dashed lines. Data of one representative experiment are shown.

Our results indicated that RelP proteins synthesize (p)ppGpp more efficiently than RelQ, because RelP can more readily bind the GDP/GTP substrate through increased rigidity of the G-Loop. Moreover, pppGpp stimulates the activity of RelQ, while it does not for RelP (Figs 2d and 3f). Therefore, we hypothesized that binding of pppGpp to the central cleft of RelQ might be translated into an increased (p)ppGpp synthesis via the G-Loop. Superimposition of the crystal structures of apo-*Bs*RelQ and pppGpp-bound *Bs*RelQ (PDB: 5DEC and 5DED^33^, respectively) allowed tracing a structurally possible path, which would connect the presence of pppGpp within the allosteric cleft of RelQ with the G-loop (Fig. 4). In short, two opposing subunits of the *Bs*RelQ tetramer are involved in coordination of one allosteric pppGpp in the central cleft^1,33^. Coordination of pppGpp leads to a displacement of Phe42, Thr44 and Asn148 by ~1–2 Å towards the cleft (Figs 4b; S2). Helix α4 comprising Asn148 follows this movement and rotates by approximately 15° in a counterclockwise manner. This movement is relayed onto helix α5 through the hydrophobic core between both helices constituted by Phe149 (α4), Leu183 and Met187 (both α5, Fig. 4c). Rotation of α5 turns Glu178 towards the G-Loop and enables formation of a salt bridge between Glu178 and Arg117 (Fig. 4c). Further contacts between α5 and the G-Loop are established between His111/Glu178 and Glu113/Gln174 (Fig. 4d).

To probe the participation of these amino acids, we replaced them by alanine and measured the (p)ppGpp synthesis of the resulting *Bs*RelQ variants in pppGpp-dependent manner (Figs 4e and S4). Variation of His111 and Glu113 does not affect stimulation of *Bs*RelQ. However, upon replacement of Gln174 and Glu178 the pppGpp-stimulatory effect is decreased and completely abolished when Arg117 is replaced (Figs 4e and S4).

Finally, we tested how the allosteric pppGpp affects the enzyme kinetic behaviour of *Bs*RelQ by determining the (p)ppGpp synthesis *Bs*RelQ in presence of different concentrations of pppGpp (i.e. 0, 2.5, 10, 25, 100, 250 µM). While addition of increasing amounts of pppGpp to *Bs*RelQ does only slightly elevate V_max_ of (p)ppGpp synthesis, the K_m_ values for the substrates GDP and GTP decrease dramatically (Figs 4f and S5). Also, *Bs*RelQ displays a less cooperative behaviour indicated by a loss of the sigmoidal shape of the v/S characteristic when pppGpp is present. It therefor appears to us that the apparent cooperativity of *Bs*RelQ rather originates from pppGpp produced during the enzymatic reaction rather than from a positive cooperativity between the four active sites of *Bs*RelQ (compare to Fig. 2a and ref.^29^). Noteworthy, at the highest concentration of pppGpp tested (i.e. 250 µM), the enzyme kinetic behavior of *Bs*RelQ is highly similar to *Bs*RelQ-H111P and *Sa*RelP.

These results show that allosteric binding of pppGpp causes structural rearrangements of *Bs*RelQ that are translated into an increased (p)ppGpp synthetase activity via an induced structural rigidity of the G-Loop.

## Discussion

Two small alarmone synthetases (i.e. RelP/SAS2 and RelQ/SAS1) are typically found together in members of the Firmicutes phylum e.g. *B. subtilis*, *S. aureus* or *L. monocytogenes*^20^. RelP and RelQ share similarities of ~50 percent on the amino acid sequence level. Our structural analysis shows that RelP and RelQ possess a highly similar (p)ppGpp synthetase domain and both establish highly similar homotetrameric complexes (Fig. 1b and c). Nevertheless, both enzymes decisively differ in their ability to produce (p)ppGpp in that RelP is much more active than RelQ (Fig. 2a). Why is that the case? Our analysis demonstrates that binding of ATP proceeds in identical fashion in RelP/RelQ proteins, because both proteins harbor an identical architecture of their ATP-coordination site (Fig. S3). However, RelP and RelQ inherently differ in their ability to coordinate the GDP and GTP substrates. This is caused by a different structural flexibility of their G-Loops. While the G-loop of RelQ is highly disordered, the equivalent region of RelP is highly ordered and can therefore readily coordinate GDP/GTP (Fig. 3). However, the activity of RelQ can be enhanced by coordination of pppGpp within the central cleft^33^. This pppGpp results in a rearrangement of helices α4 and α5 at the lateral sides of the RelQ homotetramer and, by establishing a salt bridge between Glu178 (α5) and Arg117 (G-Loop) (Fig. 4), results in a more ordered (and active) conformation of the G-Loop. The (p)ppGpp synthetase activity of the so-stimulated RelQ resembles RelP. Notably, the K_m_ values obtained for *Sa*RelP (Fig. 2a and b) and allosterically stimulated *Bs*RelQ (Figs S4 and S5) accord with the intracellular concentrations of GDP and GTP, estimated as 200–500 µM and 1–5 mM, respectively^34,35^. Under these conditions, both enzymes are highly sensitive to small changes in GDP/GTP levels. Non-stimulated *Bs*RelQ, in contrast, appears rather insensitive to changes in GDP/GTP levels because of its high K_m_ values for both substrates (Fig. 2a and b). In summary, RelP always appears as a highly active alarmone synthetase, while RelQ can switch between a passive state with low and an active (i.e. pppGpp-stimulated) state with high (p)ppGpp synthetase activity.

**Figure 5:**
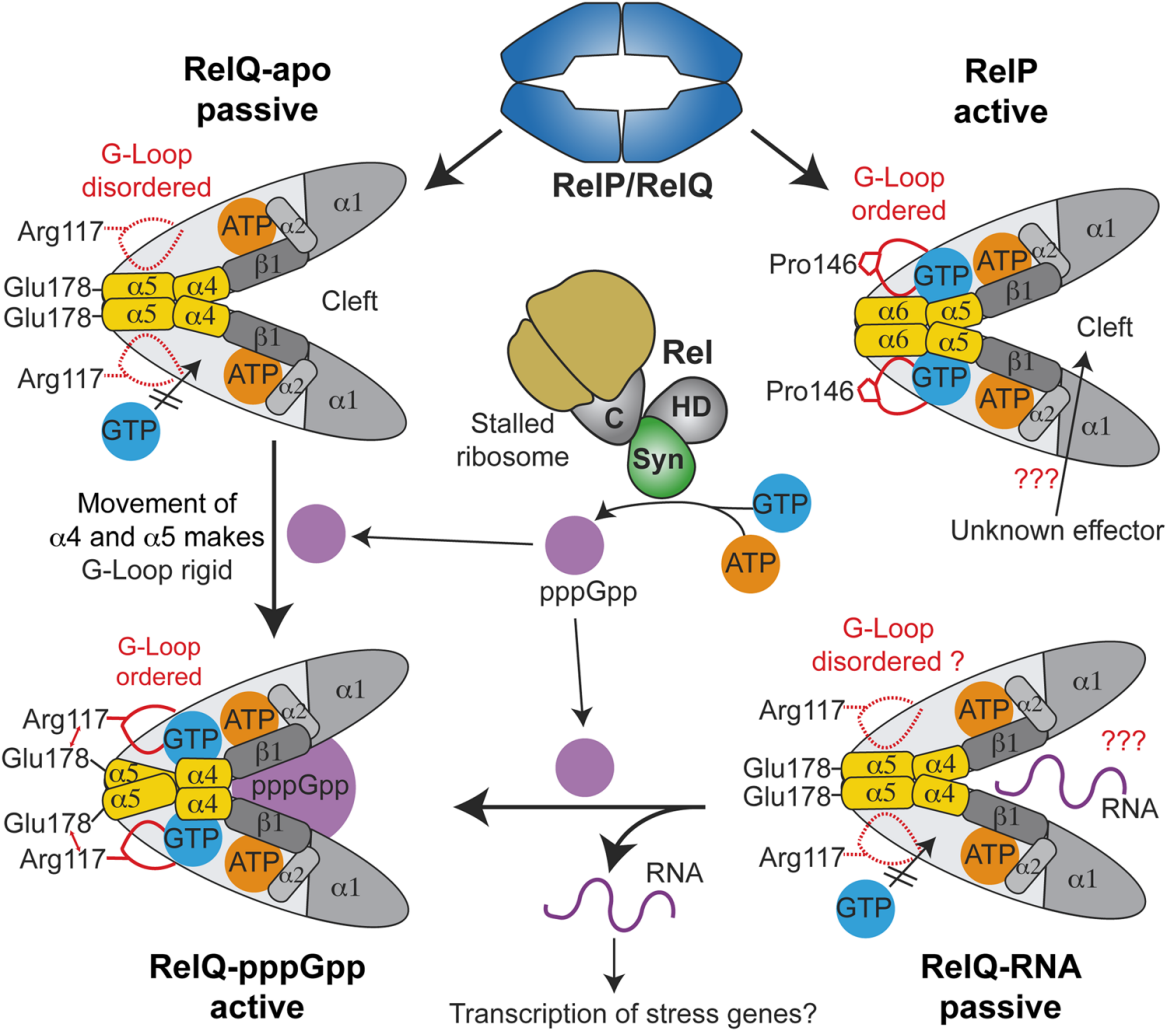
Mechanistic framework of Rel, RelP and RelQ. Three states of RelQ differing in (p)ppGpp synthetase activity are known: apo-RelQ and the RNA-bound RelQ are catalytically passive states, while RelQ bound to the alarmone pppGpp is an active (p)ppGpp synthetase. In both passive states, RelQ readily binds ATP (orange). However, GTP (blue) is only poorly coordinated, because of the disordered nature of the G-Loop. Binding of pppGpp (violet) into the allosteric cleft of RelQ results in a concerted rearrangement of α4 and α5 (yellow) that rigidifies the G-Loop, enables tight coordination of GTP and renders RelQ highly active. Such pppGpp molecules might originate from Rel’s (p)ppGpp synthetase activity, which is enhanced under conditions of amino acid starvation. In such a case, pppGpp might bind into to the unoccupied central cleft of apo-RelQ or could competitively replace an RNA molecule from the cleft as shown previously^29^. The G-Loop of RelP is always ordered enforcing the active state of RelP. Whether an RNA or any other unknown effector molecule can bind into the central cleft of RelP is not known.

Having elucidated the different properties of RelP and RelQ, we wondered how this divergence might be relevant for the bacterial cell. In our current understanding, RelQ can appear in two passive states. In the apo-state, RelQ’s central cleft is unoccupied while in the RNA-bound state a so far uncharacterized RNA^29,36^ might reside in the central cleft (Fig. 5). We suspect that RelQ is predominantly found in either of those passive states in nutrient-rich conditions, because the (p)ppGpp hydrolytic activity of Rel should keep (p)ppGpp levels below the limit of RelQ stimulation. When the microorganism is suddenly confronted with nutrient limitation, Rel will recognize and bind to stalled ribosomes. When doing so, Rel could provide the pppGpp needed to bring RelQ into its active (i.e. pppGpp-bound) state by the intricate mechanism involving helical rearrangements and loop stabilization (Fig. 5). RelQ would then simply serves as an amplifier of the stress signal given by Rel. Additionally, the RNA bound to RelQ would be outcompeted by pppGpp and might result in the transcription of stress genes. Unfortunately, it is unclear so far, which genes might be differentially regulated, as the ‘real’ RNA bound by RelQ *in vivo* still remains to be identified^29,36^. Seemingly, RelQ’s activity is intensively coupled to Rel (Fig. 5). Although experimental data for this functional link of Rel and RelQ are missing so far, the outlined scenario would provide an elegant way for an instant rise of (p)ppGpp levels dominated by the activity Rel and aided by RelQ.

RelP, in contrast to RelQ, is always a highly active enzyme that possesses all features enabling efficient (p)ppGpp synthesis, mainly an ordered G-Loop (Fig. 5). RelP should therefore not rely on the signal provided by Rel but might rather work independently. The presence of a central cleft within the tetramer of RelP nevertheless allows hypothesizing that an unknown factor might regulate the activity of RelP (Fig. 5). Noteworthy, the different activities of RelP and RelQ seem to be perfectly matched with their disparate transcriptional profiles. The switchable RelQ, predominantly transcribed during logarithmic growth^27^, can counteract a sudden nutrient limitation with the help of the Rel protein. The presence of RelP during logarithmic growth, however, might be detrimental for the microorganism. Consequently, RelP transcripts appear only during early stationary phase and in response to treatment with antibiotics, ethanol, high salt and acidic or alkalic pH stress conditions^25,37,38^. Also, RelP has been implicated in mediating inactivation of ribosomes by forming translation-inactive ribosome dimers thereby providing an elegant and fast shutdown mechanism for the bacterial metabolism^32,39^. In conclusion, our study strengthens the understanding of disparate roles of RelP/RelQ proteins and sets the stage for future investigations on this class of (p)ppGpp synthetases.

## Materials and Methods

### Cloning and mutagenesis

Genes encoding for RelP (*ywaC* and *SA2297*, respectively) were amplified from *B. subtilis* PY79 and *S. aureus* strain Newman genomic DNA by polymerase chain reaction using Phusion High-Fidelity DNA polymerase (NEB) according to the manufacturer’s manual. The *forward* primer for *SA2297* encoded a hexahistidine-tag in frame with the DNA sequence of *relP*. The *forward* primer for *ywaC* encoded a strep-tag in frame with the DNA sequence. The resulting PCR fragments were cloned into the pET24d(+) vector (Novagen) at the *Nco*I/*Xho*I restriction sites. Mutations within RelP were generated by overlapping PCR.

### Protein Production and Purification

*Escherichia coli* BL21 (DE3) (NEB) carrying the plasmids for His-tagged proteins were grown in lysogeny broth (LB)-medium supplemented with 50 µg/ml kanamycin and 12.5 g/l D(+)-lactose-monohydrate for 20 h at 30 °C. Cells were harvested by centrifugation (3500 × *g*, 20 min, 4 °C), resuspended in lysis buffer (20 mM of HEPES-Na pH 8.0, 250 mM NaCl, 40 mM imidazole, 20 mM MgCl_2_, 20 mM KCl) and lysed by two passages through the M-110L Microfluidizer (Microfluidics). After centrifugation (47850 × *g*, 20 min, 4 °C), the clear supernatant was loaded on a 1-ml HisTrap column (GE Healthcare) equilibrated with 10 column volumes (CV) lysis buffer. After washing with 10 CV of lysis buffer, the protein was eluted with 5 CV elution buffer (lysis buffer containing 500 mM imidazole). The protein was concentrated (Amicon Ultracel-10K (Millipore)) and applied to size-exclusion chromatography (SEC) on a HiLoad 26/600 Superdex 200 pg column (GE Healthcare) equilibrated in SEC buffer (20 mM of HEPES-Na, pH 7.5, 200 mM NaCl 20 mM MgCl_2_, 20 mM KCl). Protein containing fractions were pooled, concentrated (Amicon Ultracel-10K (Millipore)), deep-frozen in liquid nitrogen and stored at −80 °C. Protein concentration was determined by a spectrophotometer (NanoDrop Lite, Thermo Scientific).

*Bs*RelP was purified by a similar procedure using a 1-ml StrepTrap column (GE Healthcare). Lysis buffer without imidazole was employed for cell lysis, column equilibration and washing and elution from the column was conducted with 5 CV of SEC buffer containing 2.5 mM desthiobiotin.

### Preparation of ppGpp and pppGpp

(p)ppGpp was produced essentially as described previously^33^. In brief, 5 µM SAS1 were incubated in SEC buffer together with 10 mM ATP and 10 mM GDP for 30 min at 37 °C to produce ppGpp or together with 10 mM ATP and 10 mM GTP for 2 h at 37 °C to produce pppGpp. Afterwards, the reaction was mixed with the same volume of chloroform and centrifuged (17300 × *g*, 5 min, 4 °C). The aqueous phase was removed and the organic phase mixed with one volume of double-destilled water and centrifuged (17300 × *g*, 5 min, 4 °C). The combined aqueous phases were subjected to anion-exchange chromatography using a ResourceQ. 6-ml column (GE Healthcare) at a flow rate of 6 ml/min and the nucleotides eluted with a gradient of NaCl. Fractions containing ppGpp or pppGpp were pooled followed by addition of lithium chloride with a concentration of 1 M and four volumes of ethanol. The suspension was then incubated at −20 °C for 20 min and centrifuged (5000 × *g*, 20 min, 4 °C). The resulting pellets were washed with absolute ethanol, dried and stored at −20 °C. Quality of the so-prepared alarmones was controlled by HPLC and yielded ppGpp and pppGpp in purities of 98% and 95%, respectively.

### Kinetic analysis of RelP/RelQ

The enzyme kinetic behavior of RelP and RelQ (compare to Figs 2a, 3e, 4f and S5), were monitored by HPLC. Reactions were prepared in SEC buffer supplemented with 100 mM HEPES-Na pH 7.5 by incubating 0.2 µM protein together with 5 mM ATP and varying concentrations of GDP or GTP (i.e. 0.05, 0.1, 0.2, 0.3, 0.5, 1, 3 and 5 mM; 2 and 4 mM were included where necessary). For the analysis of pppGpp affecting the kinetic behavior of *Bs*RelQ, pppGpp was also added to the reaction in concentrations of 0/2.5/10/25/100/250 µM. Samples were taken after different time points (i.e. 2, 4, 6, 8 and 10 minutes) and stopped as follows: two volume parts of chloroform were added to the sample, thoroughly mixed for 15 seconds, kept at 95 °C for 15 seconds and flash-frozen in liquid nitrogen. While thawing, the samples were centrifuged (17300 × *g*, 30 min, 4 °C) and the aqueous phase used for analysis. HPLC measurements were conducted on an Agilent 1100 Series system (Agilent technologies) equipped with a C18 column (EC 250/4.6 Nucleodur HTec 3 µM; Macherey-Nagel). Nucleotides were eluted isocratically with a buffer containing 50 mM KH_2_PO_4_, 50 mM K_2_HPO_4_, 10 mM TPAB (tetrapentylammonium bromide) and 20% (v/v) acetonitrile and detected at 260 nm wavelength in agreement with standards. Analysis of enzymatic measurements was performed with GraphPad Prism version 6.04 for Windows, (GraphPad Software, San Diego, California, USA). The velocity of (p)ppGpp synthesis was obtained by linear regression of the amount of AMP quantified after different incubation times. Kinetic parameters (K_m_, V_max_ and the Hill coefficient (h) ± standard deviation) were obtained from the fit of the v/S characteristic according to the equation v = V_max_ S^h^/(K_m_^h^ + S^h^).

### Stimulation of RelP/RelQ by (p)ppGpp

In experiments probing the stimulatory effect of (p)ppGpp (compare to Figs 2d, 3f, 4e and S4), 0.2 µM RelP/RelQ were incubated together with 5 mM ATP and 0.25 mM GDP/GTP in presence or absence of 200 µM (p)ppGpp for 10 minutes at 37 °C. The reactions were stopped and analyzed as described above.

### Crystallization and structure determination

Crystallization was carried out at room temperature by sitting drop vapor diffusion in SWISSCI MRC 2-well plates (Jena Bioscience) with a reservoir volume of 50 µl and the drop containing 0.5 µl of protein and crystallization solution each. Crystals of *Bs*RelP were obtained from a 10 mg/ml solution after 1 week from 0.1 M CHES pH 9.5 and 30% (w/v) PEG 3000. Crystals of *Sa*RelP were obtained from a 15 mg/ml solution after 1 week in 0.1 M CHES pH 9.5 and 40% (v/v) PEG600. For crystallization of *Sa*RelP-AMPCPP, a 15 mg/ml concentrated protein solution was incubated together with 5 mM AMPCPP for 30 minutes on ice. Crystals of *Sa*RelP-AMPCPP were obtained after 2 days from 0.1 M Tris pH 8.5, 0.2 M lithium sulfate and 30% (w/v) PEG4000.

To harvest crystals, 0.5 µl of a cryo-protecting solution containing mother liquor supplemented with 20% (v/v) glycerol was added to the drop, crystals looped and flash-frozen in liquid nitrogen. Diffraction data were collected at the European Synchrotron Radiation Facility (ESRF) Grenoble, France, at beamlines ID23-1 and ID29 under laminar nitrogen flow at 100 K (Oxford Cryostream 700 Series) with a DECTRIS PILATUS 6M detector. Data were processed with XDS^40^ and CCP4-implemented SCALA^41^. Crystal structures were determined by molecular replacement (MR) employing BsRelQ (PDB: 5DEC^33^) as search model using the CCP4-implemented PHASER^41^. Structures were manually built in COOT^42^ and refined with PHENIX^43^. Figures were prepared with PYMOL (www.pymol.org).

## Accession Codes

Atomic coordinates and structure factors were deposited in the Protein Data Bank (PDB) under 6FGJ (apo-*Sa*RelP), 6FGK (apo-*Bs*RelP) and 6FGX (AMPCPP-bound *Sa*RelP).

## Electronic supplementary material

Supplementary Information
