# Supplementary material for: Structural and mechanistic divergence of the small (p)ppGpp synthetases RelP and RelQ
Source: Sci Rep. 2018 Feb 1;8:2195. doi: 10.1038/s41598-018-20634-4 (PMC5794853; doi:10.1038/s41598-018-20634-4)
Supplement: Supplementary file 1 — Supplementary Information [file 41598_2018_20634_MOESM1_ESM.pdf]

Supplementary Information for:

Structural and mechanistic divergence of the small (p)ppGpp synthetases RelP and RelQ

Wieland Steinchen[1]*, Marian S. Vogt[1], Florian Altegoer[1], Pietro I. Giammarinaro[1], Petra Horvatek[2], Christiane Wolz[2] and Gert Bange[1]*

[1]Philipps-University Marburg, LOEWE Center for Synthetic Microbiology & Department of Chemistry, Hans-Meerwein-Straße, 35043 Marburg

[2]Interfaculty Institute of Microbiology and Infection Medicine, University of Tübingen, Germany.

*Correspondence: gert.bange@synmikro.uni-marburg.de or wieland.steinchen@synmikro.uni-marburg.de

The Supplementary Information includes:

Supplementary Figures 1 – 5

Supplementary Table 1

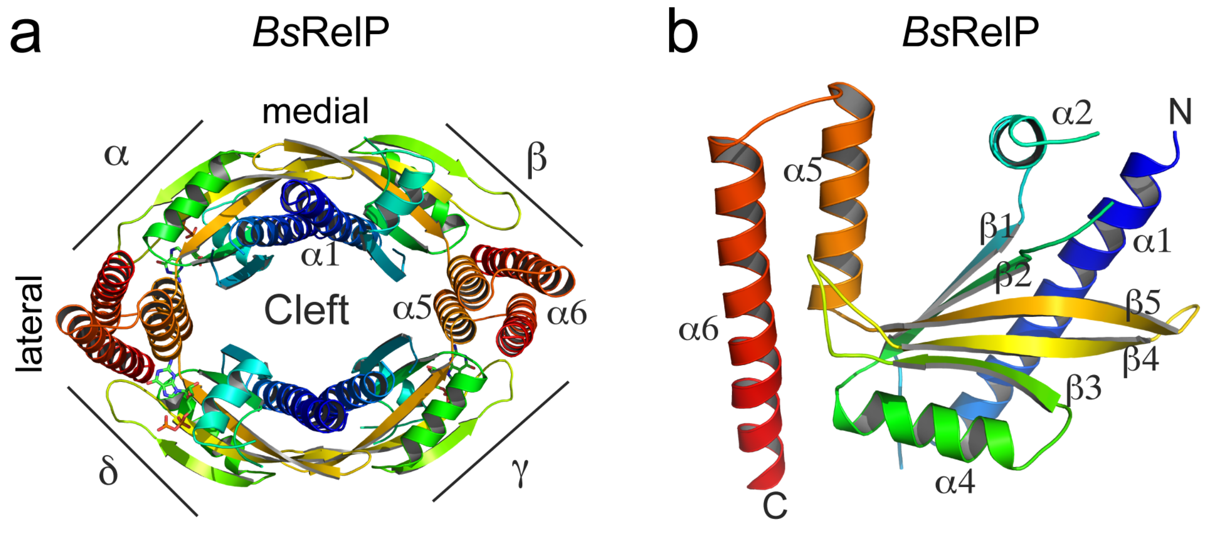

Figure. S1. The crystal structure of *Bs*RelP. (**a**) Crystal structure of the *Bs*RelP homotetramer. Each monomer (α-δ) is rainbow-colored from N- to C-terminus. (**b**) The (p)ppGpp synthetase monomer of *Bs*RelP.

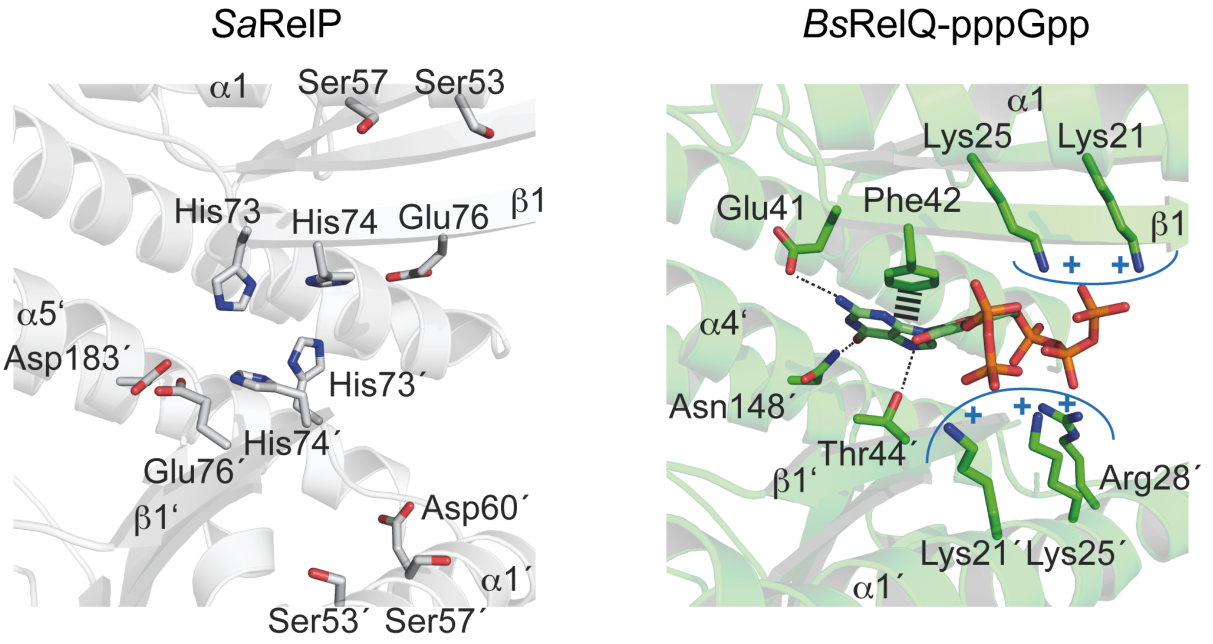

Figure. S2. Structural comparison of the central cleft of RelP and RelQ. *Sa*RelP (*Left*) and *Bs*RelQ (*Right,* PDB: 5DED) significantly differ in the architecture of their central clefts rendering *Sa*RelP unable to coordinate allosteric pppGpp.

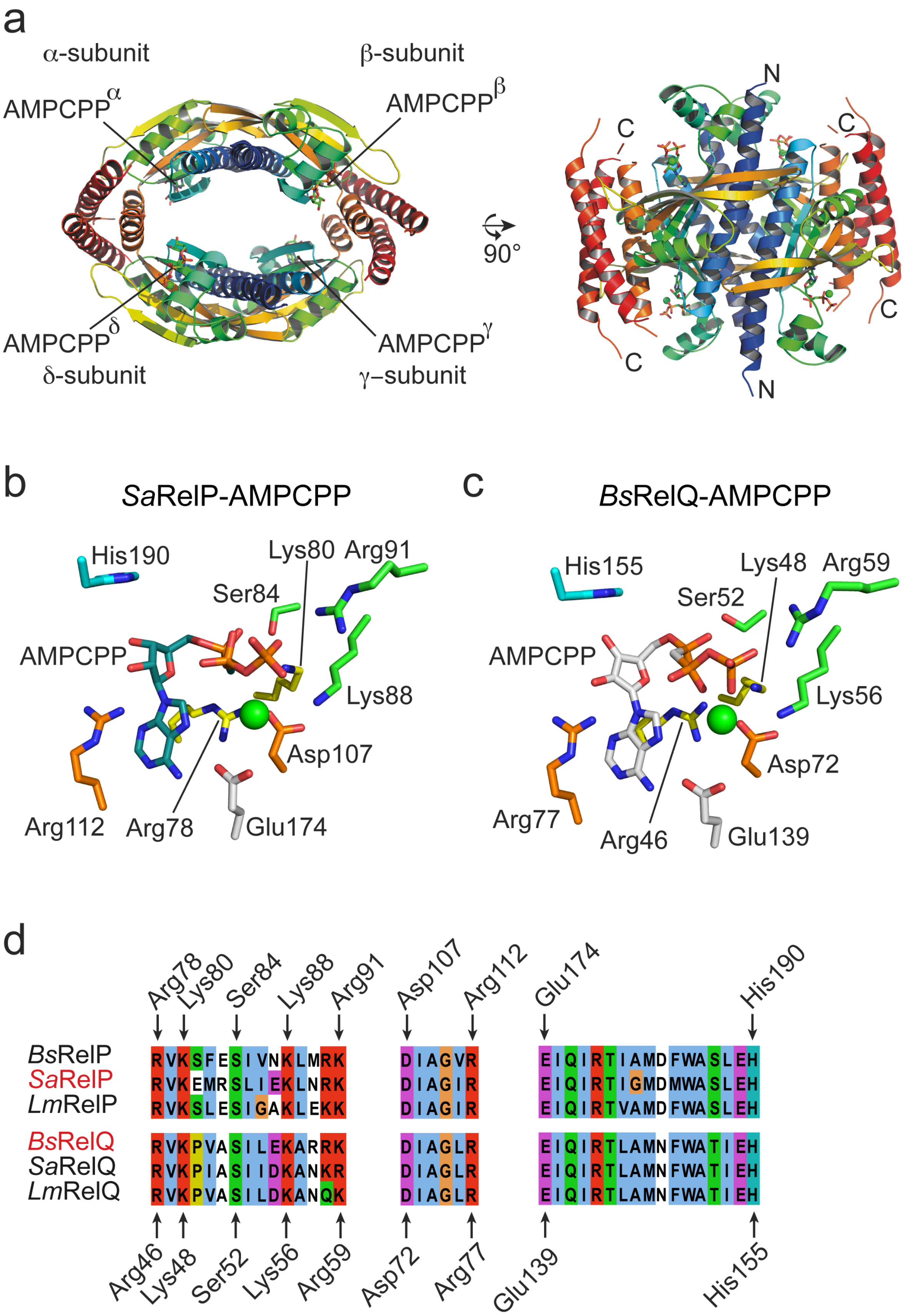
a
α-subunit
β-subunit
AMPCPP$^{\alpha}$
AMPCPP$^{\beta}$
AMPCPP$^{\delta}$
AMPCPP$^{\gamma}$
δ-subunit
γ–subunit
90°
N
C
C
C
C
N
b
*Sa*RelP-AMPCPP
His190
Lys80
Arg91
Ser84
AMPCPP
Lys88
Asp107
Arg112
Arg78
Glu174
c
*Bs*RelQ-AMPCPP
His155
Lys48
Arg59
Ser52
AMPCPP
Lys56
Asp72
Arg77
Arg46
Glu139
d
Arg78 Lys80 Ser84 Lys88 Arg91 Asp107 Arg112 Glu174 His190
*Bs*RelP RVKSFESIVNKLMRK DIAGVR EIQIRTIAMDFWASLEH
*Sa*RelP RVKEMRSLIEKLNRK DIAGIR EIQIRTIGMDMWASLEH
*Lm*RelP RVKSLESIGAKLEKK DIAGIR EIQIRTVAMDFWASLEH
*Bs*RelQ RVKPVASILEKARRK DIAGLR EIQIRTLAMNFWATIEH
*Sa*RelQ RVKPIASIIDKANKR DIAGLR EIQIRTLAMNFWATIEH
*Lm*RelQ RVKPVASILDKANQK DIAGLR EIQIRTLAMNFWATIEH
Arg46 Lys48 Ser52 Lys56 Arg59 Asp72 Arg77 Glu139 His155

Figure. S3. *Sa*RelP and *Bs*RelQ share the common ATP-binding site. (**a**) The crystal structure of *Sa*RelP bound to AMPCPP is shown in cartoon representation with each monomer (α-δ) colored in rainbow from N- to C-terminus. AMPCPP is shown as sticks. (**b**) ATP (mimicked by AMPCPP) is tightly coordinated within the active site of *Sa*RelP. The magnesium ion is shown as a green sphere. (**c**) ATP (mimicked by AMPCPP) is coordinated in identical fashion within the active site of *Bs*RelQ (PDB: 5F2V). (**d**) Amino acid sequence alignment of residues involved in ATP-binding and catalysis found in RelP and RelQ proteins from *Bacillus subtilis* (*Bs*), *Staphylococcus aureus* (*Sa*) and *Listeria monocytogenes* (*Lm*). Amino acid numberings relate to *Sa*RelP (above) and *Bs*RelQ (below).

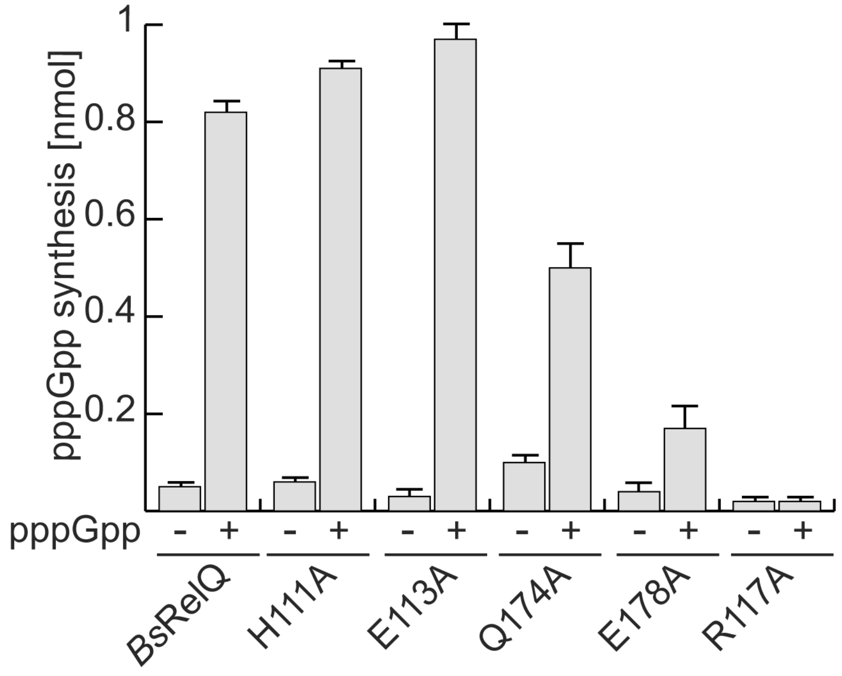

Figure. S4. Interactions between α5 and the G-Loop in *Bs*RelQ. pppGpp synthesis of *Bs*RelQ and its variants in absence (-) and presence (+) of pppGpp. Error bars indicate the SD of three independent replicates.

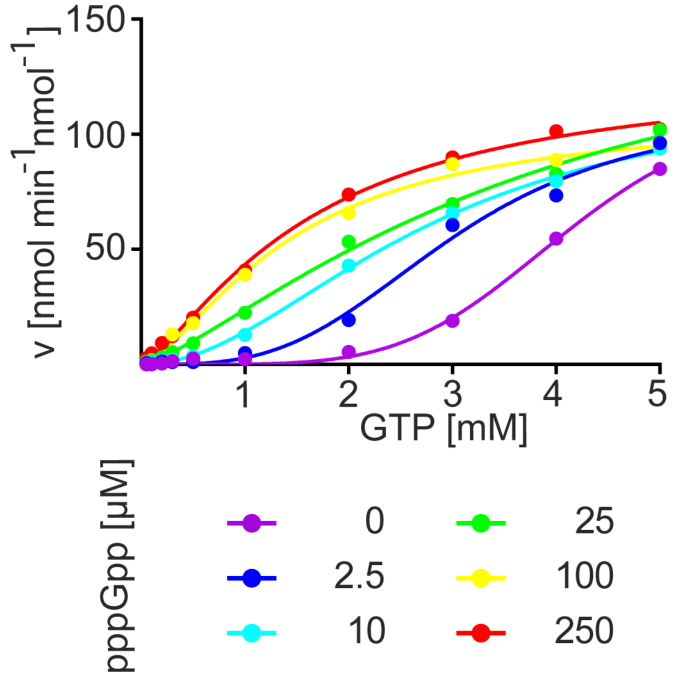

Figure. S5. pppGpp decreases the K_m for pppGpp synthesis of *Bs*RelQ. v/S characteristic of pppGpp synthesis by *Bs*RelQ in presence of different amounts of pppGpp. The velocity is given in nmol per minute per nmol *Bs*RelQ. Data of one representative experiment are shown.

Table S1. Crystallographic data collection and refinement statistics for *Staphylococcus aureus* (*Sa*) and *Bacillus subtilis* (*Bs*) RelP.

	***Sa*RelP**	***Sa*RelP - AMPCPP**	***Bs*RelP**
Data collection			
Space group	$P4_32_12$	$I4_122$	$P2_12_12_1$
Resolution (Å)	49.06 - 2.25 (2.33 - 2.25)	46.46-2.90 (3.00-2.90)	47.28 – 3.20 (3.42 – 3.20)
Unit cell parameters			
a, *b*, *c* (Å)	71.80 71.80 190.82	125.86, 125.86, 217.88	83.67 99.92 122.88
α, *β*, *γ* (°)	90, 90, 90	90, 90, 90	90, 90, 90
R_{merge}	0.0364 (0.580)	0.178 (1.234)	0.169 (0.572)
Average $I/\sigma(I)$	20.37 (2.22)	14.65 (2.19)	3.7 (0.9)
No. of total reflections	103930 (10417)	231522 (22086)	16011 (1561)
Redundancy	4.3 (4.4)	11.7 (11.4)	4.1 (4.2)
Completeness (%)	99.0 (100.0)	100.0 (100.0)	92.2 (92.7)
CC½ (%)	1 (0.85)	99.7 (79.7)	0.98 (0.72)
Refinement			
R_{work}/R_{free} (%)	19.9/24.8	19.9/23.4	25.72/31.92
No. of atoms			
Overall	3314	3328	6058
Protein	3261	3211	6058
Ligands	20	64	0
Water	33	53	0
Average *B*-factors (Å^2)			
Overall	72.00	32.26	64.6
Protein	72.12	31.23	64.6
Ligands	87.05	69.31	0.00
Water	50.71	49.70	0.00
Root-mean-square deviation			
Bond lengths (Å)	0.010	0.009	0.004
Bond angles (°)	1.26	1.36	0.74
Ramachandran plot (%)			
Favored	98.74	98.00	94.23
Allowed	1.00	1.80	5.49
Outliers	0.26	0.20	0.28
